# Multidisciplinary treatment of severe spinal deformity complicated with severe cardiopulmonary dysfunction: a case report

**DOI:** 10.1186/s13052-025-01925-9

**Published:** 2025-03-24

**Authors:** Beiyu Xu, Chao Li, Longtao Qi, Yao Zhao, Wence Wu, Chengxian Yang, Ranlv Zhu, Zhengrong Yu, Chunde Li

**Affiliations:** Department of Orthopedic/Spine Surgery, Peking University First Hospital, Beijing, 100034 China

**Keywords:** Scoliosis, Cardiopulmonary dysfunction, Halo-pelvic traction(HPT), Multidisciplinary treatment(MDT)

## Abstract

**Background:**

Severe spinal deformity (Cobb angle > 90°) often leads to cardiopulmonary dysfunction, posing significant surgical risks. We report a rare case of severe kyphoscoliosis with cardiopulmonary failure treated with non-invasive ventilation, anti-shock treatment, Halo-pelvic traction (HPT), and orthopedic surgery, emphasizing the importance of multidisciplinary cooperation.

**Case presentation:**

A 13-year-old Boy with genetic suspicion of distal arthrogryposis (Type 5D) presented with Cobb angles of 94° (scoliosis) and 69° (kyphosis), respiratory failure (PO_2_ 36.3 mmHg), and pre-shock status. A multidisciplinary team stabilized the patient using non-invasive ventilation, nutritional optimization and HPT. Post-traction correction rates reached 46.8% (coronal) and 53.6% (sagittal). Subsequent posterior spinal fusion (T1-L5) achieved 69% correction, resolving cardiopulmonary dysfunction and resulting in a highly satisfactory therapeutic outcome.

**Conclusions:**

This case illustrates a case with severe spinal deformity combined with extremely severe cardiopulmonary dysfunction and highlights the importance of multidisciplinary cooperation in high-risk pediatric patients.

## Background

Severe spinal deformity is usually defined as a Cobb angle exceeding 90° [1]. However, the patients with this condition frequently exhibit restricted thoracic and abdominal cavity volume and impaired pulmonary development, leading to multiple complications in cardiac, pulmonary and digestive functions, and generally poor nutritional status. Chronic deformity progression induces spinal stiffness, complicating corrective surgery by prolonging operative time, increasing intraoperative and postoperative hemorrhage, and elevating risks of irreversible neurological injury. Perioperative complication rates are substantially higher in these cases, rendering single-stage corrective surgery suboptimal. Preoperative optimization of pulmonary function, cardiac status, and nutritional parameters significantly mitigates surgical risks [2].

Halo-pelvic traction (HPT), though historically underutilized due to its bulkiness and sleep interference, remains clinically valuable for severe deformities. HPT provides controlled gradual traction that elongates concave-side contracted tissues, enhances spinal flexibility, expands thoracic capacity, and improves pulmonary and digestive function. These biomechanical and physiological adaptations reduce corrective surgery complexity, operative duration, and blood loss.

Here, we present a critical case of severe spinal deformity with cardiopulmonary failure managed through multidisciplinary protocols based on the literature. This exemplifies the paradigm of collaborative perioperative care to minimize complications.

## Case presentation

A 13-year-old male presented with an 8-year history of progressive thoracolumbar deformity and 2-year progression of exertional dyspnea and cyanosis. Upon admission, the patient was being transported in a wheelchair. His intelligence is normal. There is no consanguinity in the parents and no history of any neurological disorders, genetic conditions, or early deaths in either parents’ family.

**Fig. 1:**
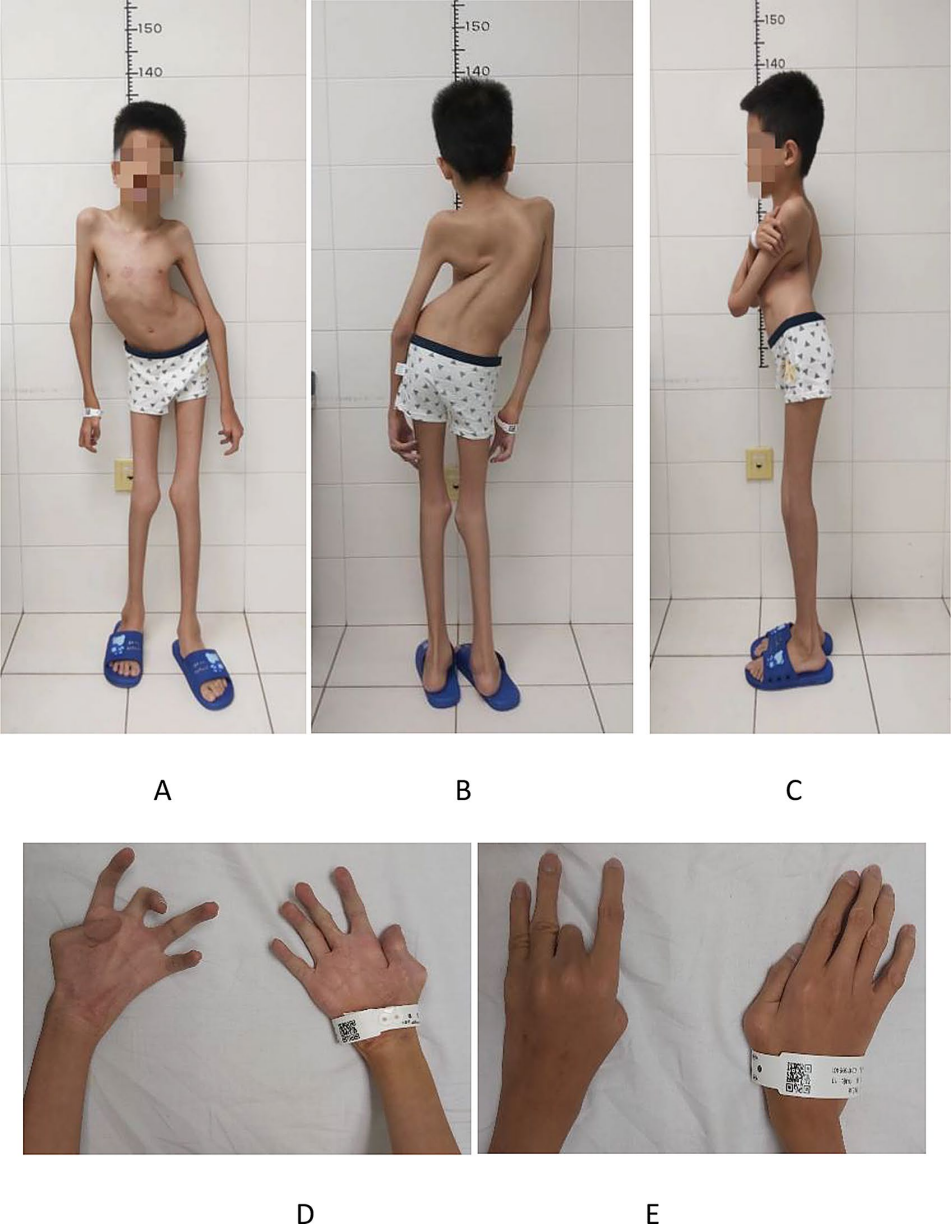
Appearance of the patient. (**A**, **B**, **C**) Anterior, posterior, and lateral appearance of the patient. (**D**, **E**) Photographs of joint deformity of hands

Admission findings included nasal oxygen (2 L/min), tachypnea (26 breaths/min), low blood pressure(75/65 mmHg) and hypoxemia (SpO_2_ 65% on room air). Anthropometrics revealed severe malnutrition: height 130 cm, weight 20 kg, BMI 11.8 kg/m^2^. Physical examination demonstrated thoracolumbar kyphoscoliosis, pectus deformity, and upper limb arthrogryposis (Fig. 1). Neurological examination was unremarkable.

Laboratory studies showed hemoglobin 92 g/L (normal: 120–160), albumin 29 g/L (35–50), hs- CTNI 44.1 ng/L (< 15), and BNP 392 pg/mL (< 100). Arterial blood gas analysis (ABG) showed FiO_2_ of 21%, pH of 7.345, PCO_2_ of 70.2 mmHg, PO_2_ of 36.3 mmHg, and lactic acid of 1.5 mmol/L, indicating severe respiratory failure(Type II).

Ultrasonic cardiogram revealed a left ventricular ejection fraction of 60.2%, enlargement of the right atrium and right ventricle, pulmonary artery dilation, moderate tricuspid regurgitation, and PASP(Pulmonary arterial systolic pressure) of 91 mmHg. Pulmonary function tests showed a maximal voluntary ventilation (VC MAX) of 0.34 L, forced expiratory volume in 1 s (FEV1) of 0.32 L, forced vital capacity (FVC) of 0.34 L, FEV1% of 19.5%, and FEV1/FVC ratio of 91.8%, indicating severely restrictive ventilatory dysfunction.

**Fig. 2:**
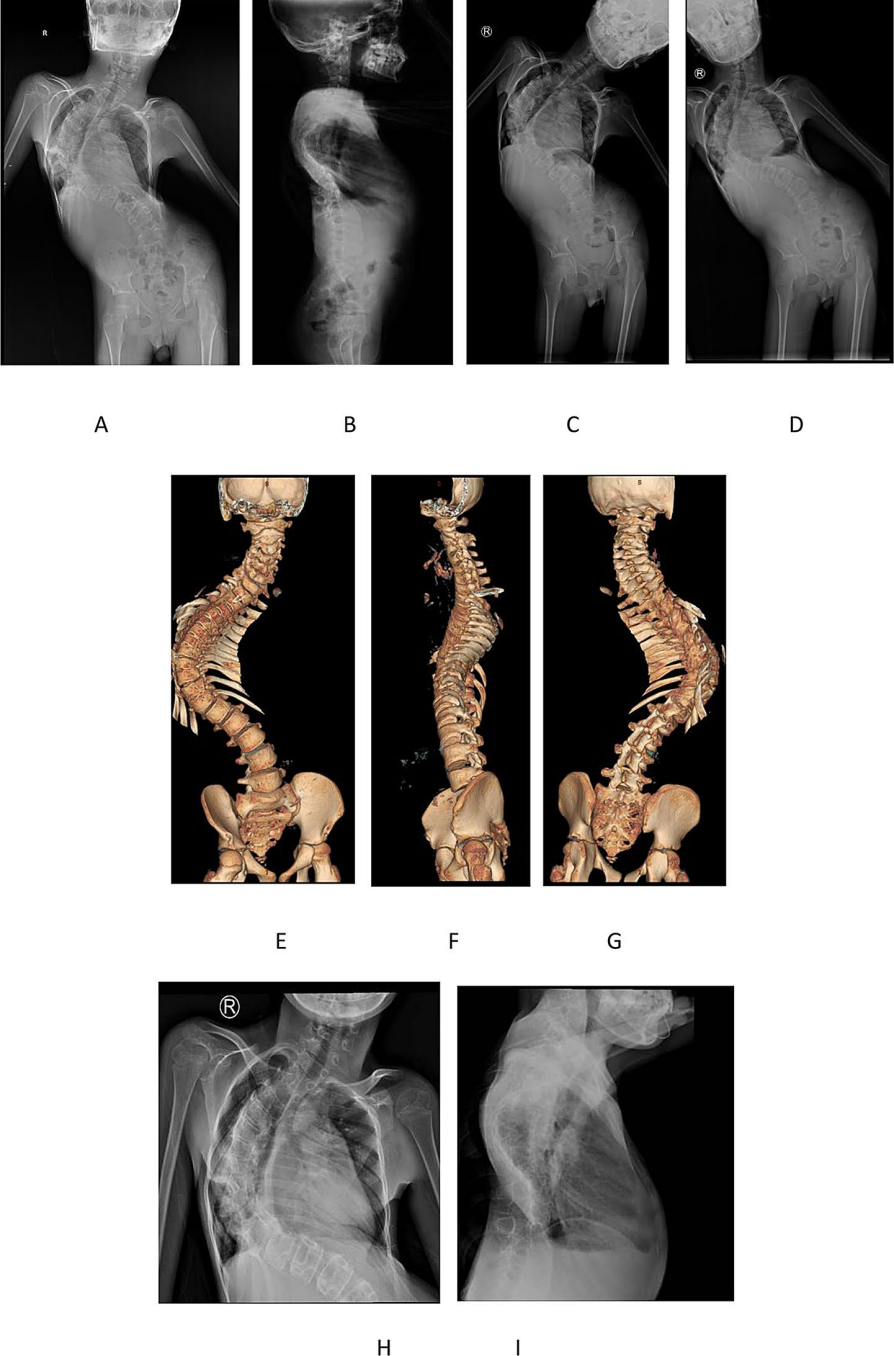
X-ray, CT images of the patient (**A**, **B**) The anteroposterior and lateral view of spinal X-ray. (**C**, **D**) The left and right bending view of spinal X-ray. (**E**, **F**, **G**) The three-dimensional CT images of spine. (H, I) The anteroposterior and lateral view of chest X-ray

Genetic analysis identified an ECEL1 variant (c.2152–15 C > A) suggestive of distal arthrogryposis type 5D. Radiographic and CT images of the spine and chest X-rays (Fig. 2) revealed severe imbalance and stiffness in the coronal and sagittal positions of the spine, shoulder imbalance, and pelvic tilt. The spinal deformity was compressing the right thoracic cavity, leading to severe incomplete inflation of the right lung.

### Treatment process

Upon admission, the patient had severe hypoxia, CO_2_ retention, pre-shock status(shock index of 2.1), and extremely unstable vital signs. The primary goal of treatment was to stabilize the patient’s vital signs, improve respiratory failure and shock status.

#### Phase 1: Stabilization

Multidisciplinary intervention targeted cardiorespiratory failure and shock with the departments of respiratory medicine, cardiology, pediatrics, anesthesia and the intensive care unit(ICU). The patient’s anesthesia classification according to the American Society of Anesthesiologists (ASA) was grade IV and indicated a constant threat to life. The patient was urgently transferred to the Respiratory Intensive Care Unit (RICU) for non-invasive ventilation support to improve ventilation. Intravenous fluid was administered through an open vein to correct the patient’s shock status. Additionally, a sputum ejection machine was used to assist in the clearance of phlegm, and second-generation cephalosporin was administered to prevent respiratory tract infections.

To aid in the patient’s recovery, the rehabilitation department was invited to guide comprehensive lung function training, including deep inhalation training, diaphragmatic breathing, and respiratory muscle strengthening exercises. The patient’s Nutrition Risk Screening (NRS) score was 4, indicating a high risk of malnutrition. Therefore, the nutrition department was consulted to develop a personalized nutrition plan, including oral nutritional supplements such as homogenized diet and whey protein powder. Recombinant human erythropoietin injection was administered by injection, along with oral iron supplements to improve the patient’s anemic condition. After 3 weeks of non-invasive ventilator support and comprehensive multidisciplinary interventions, the patient’s vital signs stabilized, and significant improvements were observed in cardiac and pulmonary functions (Table 2).

#### Phase 2: HPT

**Fig. 3:**
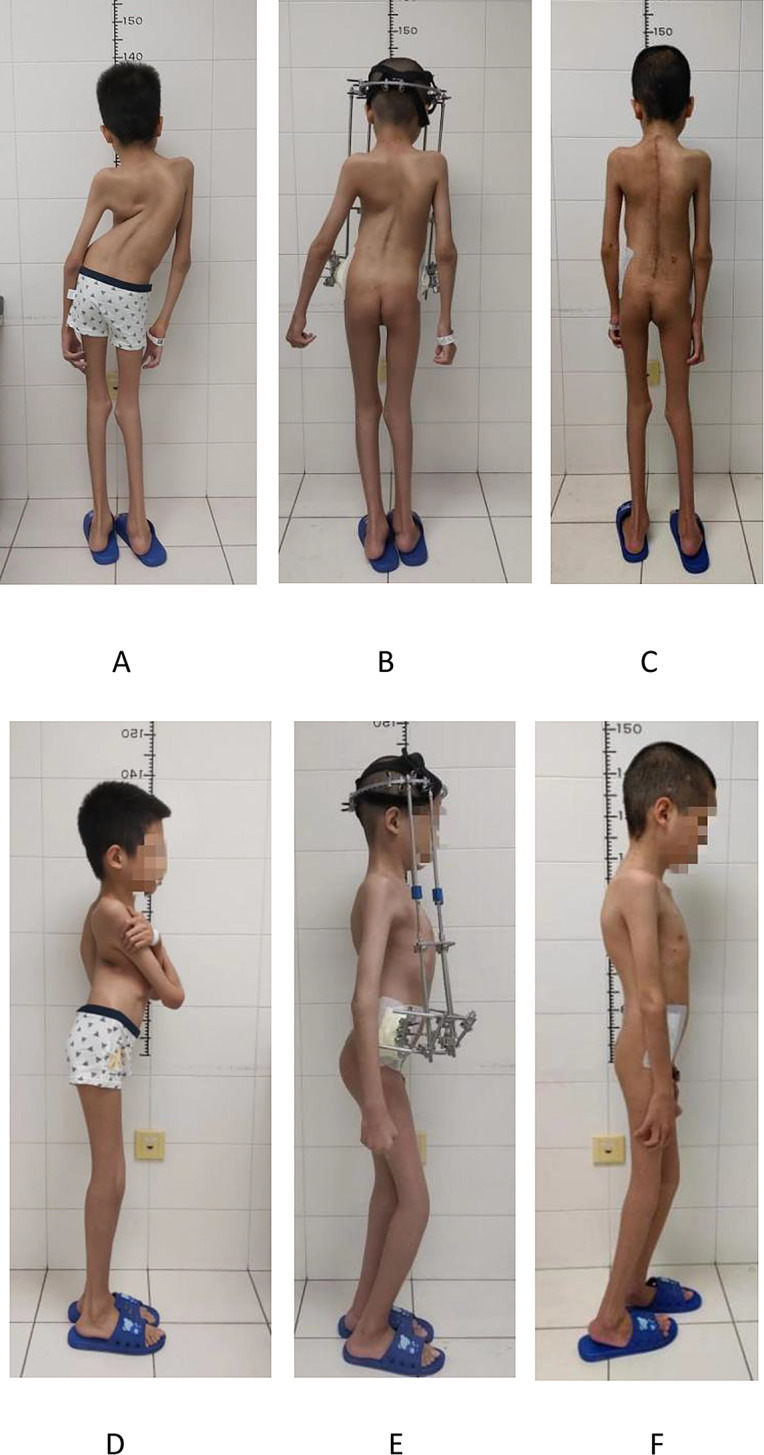
The appearance before and after treatment. **(A)** The posterior appearance before surgery. **(B)** The posterior appearance after traction. **(C)** The posterior appearance 1 week after surgery. **(D)** The lateral appearance before surgery. **(E)** The lateral appearance after traction. **(F)** The lateral appearance 1 week after surgery

To improve the compression state of the chest, reduce the stiffness and severity of the spinal deformity, a modified HPT was used in this case, with four traction rods placed on the anterolateral side of the trunk, and a front half-ring pelvic halo was used to achieve sustained traction for 24 h in one day(Fig. 3).

**Fig. 4:**
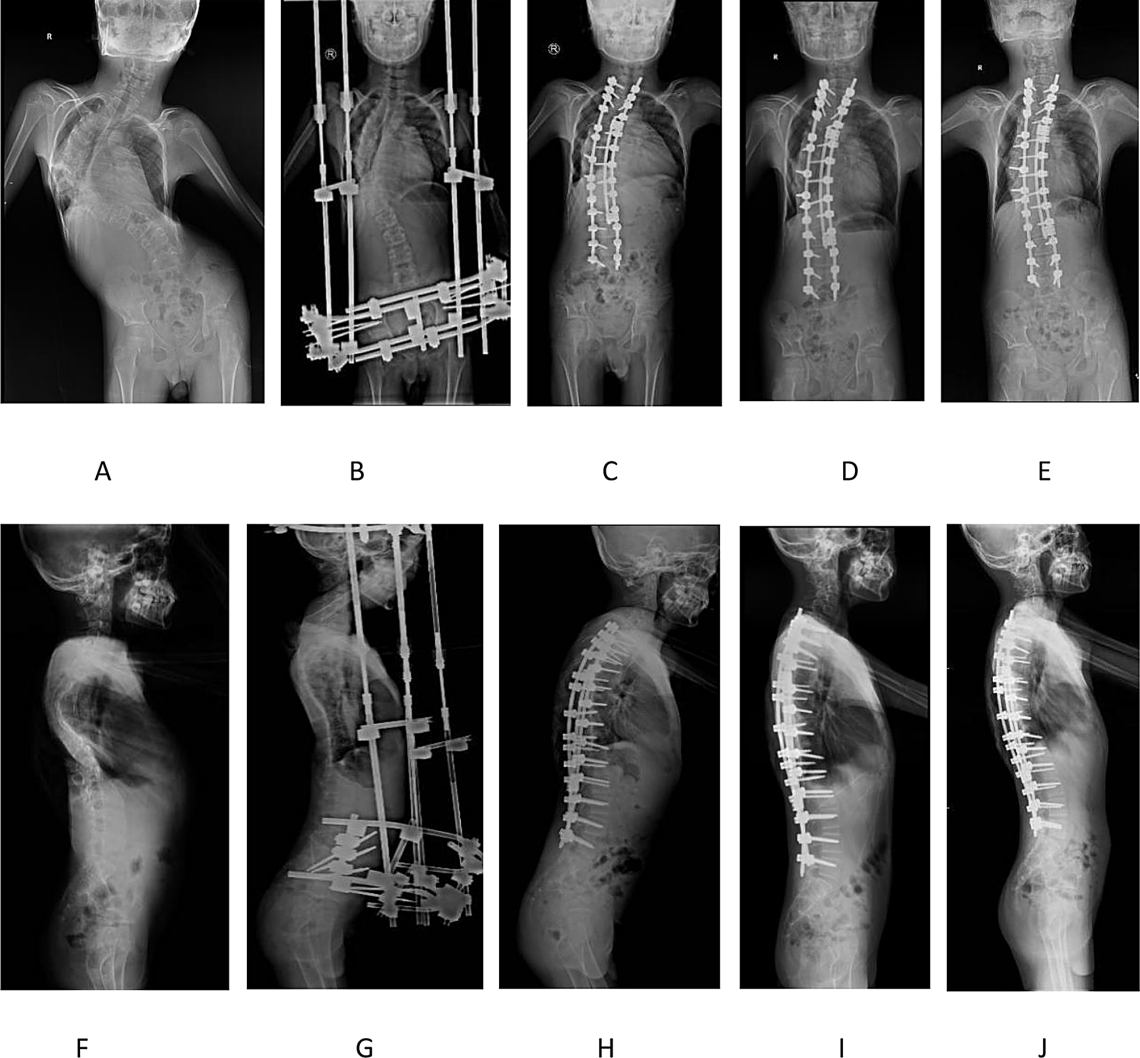
The appearance before and after treatment. **(A)** The anteroposterior view of spinal X-ray before surgery. **(B)** The anteroposterior view of spinal X-ray after traction. **(C)** The anteroposterior view of spinal X-ray 1 week after surgery. **(D)** The anteroposterior view of spinal X-ray 1 month after surgery. **(E)** The anteroposterior view of spinal X-ray 1 year after surgery. **(F)** The lateral view of spinal X-ray before surgery. **(G)** The lateral view of spinal X-ray after traction. **(H)** The lateral view of spinal X-ray 1 week after surgery. **(I)** The lateral view of spinal X-ray 1 month after surgery. **(J)** The lateral view of spinal X-ray 1 year after surgery

After 5 weeks of continuous traction, the severe imbalance in the coronal and sagittal positions of the spine was significantly corrected (Figs. 3 and 4; Table 1). The coronal spinal correction rate was 46.8% and the sagittal spinal correction rate was 53.6%, and the patient’s height increased by 10 cm. Furthermore, the pulmonary function and cardiac function improved significantly (Table 2). The patient’s anesthesia classification was reassessed as grade II (ASA).

**Table 1:** The results of spinal data about the treatment

	Preoperative	Post-HPT	1 week after surgery	1 month after surgery
Scoliosis Cobb Angle(°)	94	50	29	29
Kyphotic Cobb Angle(°)	69	32	21	18
Perpendicular distance of S1 and C7(cm)	7.7	1.9	−2.0	−1.0
Perpendicular distance of S1 and convex point (cm)	16.1	7.9	3.2	3.1
Height difference of shoulders(cm)	2.6	0.5	0.2	0.2
Height difference of crista iliaca(cm)	3.3	2.2	0.9	0.9
Inclination of pelvis(°)	14.8	7.8	3.5	3.5
Height(cm)	130	140	141	141

**Table 2:** Comparison of cardiopulmonary function data before and after treatment

	Preoperative	Post-HPT	Postoperative
VC MAX(L)	0.34	0.52	0.60
FEV1(L)	0.32	0.45	0.52
FVC(L)	0.34	0.52	0.53
ABG-PO_2_(mmHg)	36.30	72.80	79.70
ABG-PCO_2_(mmHg)	70.20	46.90	44.50
ABG-SO_2_(%)	64.90	94.70	95.40
PASP(mmHg)	91	18	17
hsCTNI(ng/L)	44.10	2.10	1.90
BNP(pg/ml)	392	66	25
Hb(g/L)	92	125	127

#### Phase 3: Correction surgery

The patient underwent a posterior spinal correction and bone graft fusion surgery (T1-L5) and thoracoplasty. A total of 27 pedicle screws were implanted, and a portion of the ribs on the convex side (T8-T10) was resected and retightened to the titanium rod. Domino linking system was used on the concave side to enhance stability. The surgery lasted for 5 h, with a blood loss of 600 mL and autotransfusion of 400 mL. To enhance the accuracy of screw placement, O-arm navigation was utilized, and intraoperative neurophysiological monitoring(IONM) was performed to monitor the patient’s nerve function aligning with the Italy’s protocol. The procedure was successful, and there were no apparent postoperative complications. The coronal and sagittal balance of the spine were further improved (Figs. 3 and 4; Table 1). Notably, pelvic tilt was significantly corrected, resulting in an overall correction rate of 69.1% in the coronal plane and 69.6% in the sagittal plane. Furthermore, there was a significant improvement in cardiovascular and pulmonary function (Table 2).

At 1-month follow-up, radiographic evaluation demonstrated maintained integrity of the internal fixation with progressive improvement in sagittal alignment (Fig. 4). The patient exhibited resolution of cyanosis and hypoxemia, achieving room-air SpO_2_ of 95% during ambulation. Vital signs stabilized at blood pressure 95/65 mmHg, heart rate 85 bpm, and respiratory rate 14/min. Physical capacity improved markedly, evidenced by 2 kg weight gain and return to baseline activities. Echocardiography revealed left ventricular ejection fraction of 66.4% with PAPS of 17 mmHg.

At 1-year follow-up, imaging confirmed stable instrumentation and progressive spinal fusion. The patient still had growth potential post-surgery. Annual cardiopulmonary surveillance was recommended until skeletal maturity.

## Discussion and conclusions

Severe spinal deformities impair thoracic capacity, potentially culminating in respiratory failure [3, 4]. While corrective surgery remains the definitive treatment for cardiopulmonary restoration [5, 6], this patient’s initial presentation with hypoxic respiratory failure (PaO_2_ 58 mmHg), hypercapnia (PaCO_2_ 62 mmHg), ASA IV status, and early shock manifestations constituted absolute surgical contraindications [7].

The European Spine Society guidelines emphasizes preoperative cardiopulmonary optimization and staged traction to reduce surgical risks, particularly in pediatric populations [3]. Non-invasive ventilation can effectively improve gas exchange and reduce CO_2_ retention. Our implementation of non-invasive ventilation achieved rapid resolution of hypercapnia (PaCO_2_ reduction from 62 to 41 mmHg) and normalization of pulmonary hypertension (PASP from 57 to 17 mmHg), suggesting secondary cardiac dysfunction. Concurrent nutritional rehabilitation with targeted supplementation (including recombinant erythropoietin and iron therapy) corrected the patient’s negative nitrogen balance and iron-deficiency anemia (Hb 78 to 121 g/L) [8].

HPT can relax the contracted tissues on the concave side of the spine, increase the intervertebral, interlaminar space and facet joint space, thereby reducing spinal stiffness and the difficulty of corrective surgery [9, 10]. Additionally, HPT can predict the maximum tolerable correction angle of the spine based on the exacerbation and relief of corresponding neurological symptoms, making it an efficient experimental treatment measure [11]. Ilyas [12]reported that that preoperative HPT not only reduced the severity of spinal deformities but also decreased the incidence of complications. Yu [13]reported that preoperative HPT can correct deformities effectively and safely, avoiding the serious complications associated with direct single-stage corrective surgery.

Moreover, the modified HPT allowed for continuous traction for 24 h in one day, achieving greater correction efficiency and lower complication rates, and ensuring better patient compliance [9]. Our HPT protocol mirrors the Gradual Correction Protocol used in Turin and Italy, which reduces intraoperative blood loss by 30%. In contrast to France’s reliance on VEPTR for younger patients, HPT offers a cost-effective alternative suitable for resource-limited settings, including rural Italian hospitals [14, 15]. The multi-disciplinary strategy reduced surgical risk from ASA IV to II, enabling successful posterior instrumentation with 11 cm height restoration, and no severe complications occurred during or after the surgery. The combination of preoperative HPT and corrective surgery resulted in significant correction of the coronal and sagittal plane of the spine, resulting in a noticeable improvement in appearance. The patient resumed schooling and social activities at 1 month follow-up, with the help of integrated rehabilitation programs in pediatric care.

Through a multidisciplinary approach, meticulous preoperative evaluation, respiratory and nutritional support, and standardized and reasonable surgical planning, the patient’s life was saved and the challenging spinal corrective surgery was completed successfully. HPT requires minimal ICU support, making it viable in regions lacking advanced facilities. This aligns with WHO recommendations for adaptable spinal care in low-resource areas. So the one-stage HPT combined with two-stage corrective surgery are a safe and effective procedure for severe spinal kyphoscoliosis with severe cardiopulmonary dysfunction.

## Data Availability

Not applicable.

## Abbreviations

HPT: Halo-pelvic traction
MDT: Multidisciplinary treatment
BMI: Body mass index
hsCTNI: High-sensitivity cardiac troponin I
Hb: Hemoglobin concentration
PASP: Pulmonary Arterial Systolic Pressure
BNP: Brain natriuretic peptide
ABG: Arterial blood gas analysis
VC MAX: Maximal voluntary ventilation
FEV1: Forced expiratory volume in 1 s
FVC: Forced vital capacity
ASA: The American Society of Anesthesiologists
RICU: Respiratory Intensive Care Unit
NRS: Nutrition Risk Screening
IONM: Intraoperative neurophysiological monitoring
WHO: World Health Organization
VEPTR: Vertical Expandable Prosthetic Titanium Rib
